# Isotope-edited ESEEM: A new method for probing copper binding sites in neurodegenerative proteins

**DOI:** 10.1016/j.jbc.2026.113310

**Published:** 2026-07-01

**Authors:** Glenn L. Millhauser, Kevin Singewald, Amanda Smart, Francesca A. Pavlovici

**Affiliations:** 1 Department of Chemistry and Biochemistry, University of California Santa Cruz, Santa Cruz, California, USA; 2 Department of Pathology, University of California, San Diego, California, USA

**Keywords:** Alzheimer’s disease, amyloid-beta, copper, EPR, intraneuronal amyloid-beta, prion disease, prion protein, segmental isotope labeling, sortase-mediated ligation, ternary complex

## Abstract

The interactions between intrinsically disordered domains in neurodegenerative proteins are often stabilized by the inclusion of physiologic metal ions such as copper or zinc. Characterizing the metal ion coordination environment in such cases is critical for assessing the stability and organization of these relevant protein–protein interactions but is challenging given the lack of regular molecular order of the relevant protein domains. We recently developed a new pulsed electron paramagnetic resonance approach that takes advantage of electron spin echo envelope modulation (ESEEM) with selective ^15^N isotope labeling. Histidine side chains with ^14^N or ^15^N coordinated to Cu^2+^ give distinct, fully resolvable signals in both ESEEM and its two-dimensional companion method HYSCORE. The approach was applied to the cellular prion protein, PrP^C^, to investigate how its otherwise neurotoxic, disordered N-terminal domain is regulated through a Cu^2+^ linkage to its globular C-terminal domain. Sortase-mediated ligation created an expressed murine prion protein with segmental ^15^N-labeling exclusive to the N-terminal domain. Isotope-edited ESEEM identifies the specific His residues from both domains that participate in this essential regulatory process. Extending this approach to hetero-protein complexes focused on how Cu^2+^ may facilitate the interaction between PrP^C^ and the Aβ peptide, a dominant component of senile plaques in Alzheimer's disease. Combining ^15^N-labeled PrP^C^ with unlabeled Aβ shows that both proteins simultaneously coordinate Cu^2+^ with Aβ contributing a single His side chain. Collectively, this review highlights isotope-edited ESEEM as an effective method for achieving residue-specific insights into metal coordination within structured and disordered protein complexes.

Prevalent neurodegenerative diseases arise from the accumulation and subsequent plaque formation of specific peptides or proteins. For example, the early stages of Alzheimer’s disease (AD) are marked by the accumulation of the 40 to 43 residue amyloid β (Aβ) peptide resulting in senile plaques, followed by intracellular tau inclusions leading to neurofibrillary tangles and neuronal death (1, 2, 3, 4). A consequence of AD and other neurodegenerative diseases is the redistribution of physiologic metal ions in the brain (5, 6). Early studies found that AD leads to a tissue region-dependent reduction of CNS copper but an increase in iron and zinc (7, 8). However, animal studies (5, 9) and *postmortem* human tissue analyses (6, 10, 11) find that copper accumulates in the Aβ-rich senile plaques. Similarly, in prion diseases, which are caused by the misfolding and aggregation of a normal cellular prion protein (PrP^C^) into a harmful, infectious form called PrP-scrapie (PrP^Sc^), elevated levels of copper and zinc are observed at the PrP^Sc^ aggregation sites (12). Several outstanding reviews provide detailed discussions of the potential role for copper in neurodegenerative diseases (6, 13, 14, 15, 16).

Both Aβ (17, 18, 19, 20) and PrP^C^ (21, 22, 23, 24, 25, 26, 27, 28) are high-affinity copper binding proteins, as is α-synuclein (29, 30, 31), the intracellular protein that causes Parkinson’s disease. These proteins interact preferentially with Cu^2+^, the dominant extracellular copper species (32). However, characterization of the specific Cu^2+^ binding modes is complicated by the fact that each protein’s putative binding sites are in flexible, intrinsically disordered regions (IDRs). It’s not surprising, therefore, that characterization of the copper binding affinity, stoichiometry and coordination mode(s) for Aβ and PrP^C^ have led to disparate results. For example, K_d_ measurements of the Aβ-Cu^2+^ complex range from 10 pM to 100 nM, a range covering four orders of magnitude (16, 33). Despite these uncertainties, if copper, specifically Cu^2+^, plays a role in neurodegeneration, for example by predisposing monomeric peptides to form plaques or by conferring deleterious redox activity within these plaques, then characterizing its affinity and coordination modes is essential.

Our interests focus on PrP^C^, the protein responsible for mad cow disease, chronic wasting disease, which continues to spread among cervids in the Rocky Mountain regions, and the human diseases kuru and Creutzfeldt-Jakob disease (34). The ∼210-residue protein avidly takes up Cu^2+^, an interaction important for regulating inherent PrP^C^ toxicity (35, 36, 37, 38, 39). PrP^C^ also plays a central role in AD by functioning as a receptor for both monomeric and oligomeric Aβ (40), the latter of which is designated Aβ_o_ (41, 42). Monomeric Aβ binding to PrP^C^ results in the translocation of Aβ through endocytosis, possibly leading to its intracellular accumulation (40). Aβ_o_ binding to PrP^C^ is implicated in transmembrane signaling ultimately driving tau phosphorylation and intraneuronal aggregation (43). Both processes are active in the early stages of AD (44). Moreover, PrP^C^ is found enriched in amyloid plaques of AD patients (45) and promotes the formation of Aβ aggregates (46).

This review is aimed at new electron paramagnetic resonance (EPR) methods for revealing how Cu^2+^ regulates PrP^C^ toxicity and, in addition, facilitates the protein’s interaction with Aβ. We will briefly discuss current models of PrP^C^- and Aβ-Cu^2+^ binding derived from EPR and complementary methods, such as affinity studies, optical spectroscopy and X-ray absorption. Next, taking advantage of protein expression with uniform ^15^N labeling, along with segmental labeling using sortase-mediated ligation (47), we describe how the distinct pulsed EPR signals of ^14^N and ^15^N resolved through electron spin echo envelope modulation (ESEEM) (48) are used to unambiguously assign site-specific histidine residues in the Cu^2+^ coordination environment. We then examine how PrP^C^ interacts with Aβ through simultaneous interactions at a copper center. This review will demonstrate how advanced EPR techniques, isotope-edited ESEEM, resolves longstanding ambiguities in copper coordination.

## Residues implicated in Cu^2+^ binding to the prion protein and the Alzheimer’s Aβ peptide

**Figure 1:**
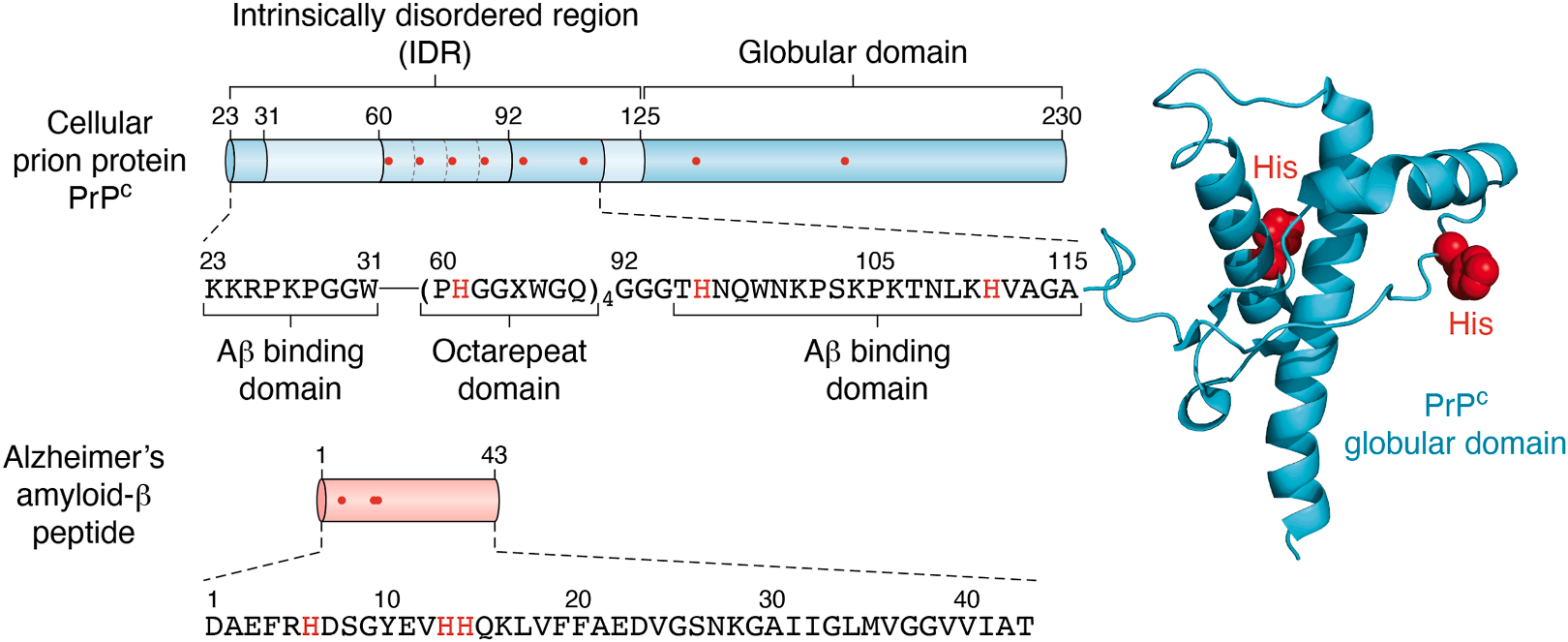
**Sequences of the cellular prion protein, PrP^C^ (*top*), and the Alzheimer’s Aβ peptide (*bottom*).** PrP^C^ possesses two domains, an IDR from residue 23 to 125 (sequence to residue 115 shown) and a predominantly helical globular domain from 126 to 230. The locations of relevant histidines in both polypeptides, implicated as possible Cu^2+^ binding sites, are indicated by *red dots*. The PrP^C^ globular domain has two His residues proposed to serve as Cu^2+^ mediated anchor points bringing the two domains together, regulating the protein’s toxicity. PrP^C^, cellular isoform of prion protein residues 23 to 230; Aβ, amyloid-β peptide; IDR, intrinsically disordered region.

Sequences for PrP^C^ and Aβ are shown in Figure 1. PrP^C^ possesses two independent domains: an IDR from residue 23 to 125 and a predominantly helical globular domain from 126 to 230 (mouse sequence after removal of the N-terminal and C-terminal signal peptides) (37, 49). Original EPR experiments identified multiple copper binding modes, depending on the ratio of Cu^2+^ to protein (23). At a low Cu^2+^:PrP^C^ ratio, Cu^2+^ is coordinated primarily by His imidazoles in the octapeptide repeat (OR) domain, a segment characterized by four His-containing repeats of the eight-residue PHGGXWGQ sequence (where X in repeats 2 and 3 represents either Gly or Ser depending on species). With additional equivalents, Cu^2+^ is also taken up by the non-OR His residues at 95 and 110 (50). Interestingly, recent work with transgenic mice finds that mutation of His95 to Tyr results in spontaneous prion disease, spongiform degeneration, and protease-resistant PrP accumulation consistent with prion disease (51). At high Cu^2+^:PrP^C^ ratios and pH > 6.5, the OR domain saturates with each repeat taking up a single Cu^2+^ equivalent, coordinated by His and backbone Gly amide nitrogens (23, 25, 26, 27). Finally, there are His residues at positions 139 and 176 in the globular domain, also implicated in copper binding by NMR experiments (39).

The 40 to 43 residue Aβ peptide (Fig. 1) is a cleavage product resulting from β- and γ-secretases that act to release the Amyloid Precursor Protein (APP) ectodomain from the extracellular membrane surface (1). Whether Aβ itself possesses physiological function remains an area of investigation. However, there is broad consensus that Aβ takes up Cu^2+^ with high affinity (16, 17, 20, 52). The interaction has been carefully investigated by a range of methods including electrochemical titrations (53), UV-Vis Absorption spectroscopy (54), NMR, continuous wave EPR (17), pulsed EPR (55), X-ray absorption and extended X-ray absorption fine structure spectroscopy (19, 56) and density functional theory calculations (57). The emerging model proposes an equatorial coordination environment composed of two His imidazoles, the N-terminal amine and an oxygen, which could come from the Asp1 side chain or backbone carbonyl (58). Common to both PrP^C^ and Aβ, of course, is the critical role of histidine, with its unique capacity for participating in the formation of high affinity Cu^2+^ binding sites (59, 60).

## ESEEM–A unique probe of IDR-Cu^2+^ interactions

**Figure 2:**
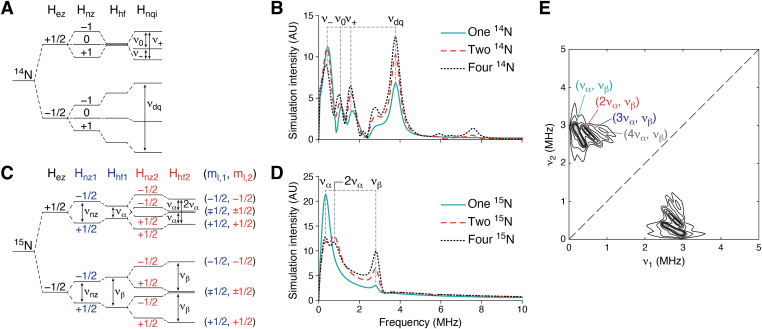
**Simulated 3-pulse ESEEM and HYSCORE spectra demonstrating the distinct signals from ^14^N and ^15^N His coordinated to Cu^2+^.***A*, the electron spin energy level diagram for ^14^N His, indicating the low frequency quadrupole transitions ν_0_, ν_−_ and ν_+_, along with a higher frequency “double quantum” transition, ν_dq_, at approximately 4.0 MHz. *B*, Cu^2+^ ESEEM spectra with one, two, and four coupled ^14^N His showing diagnostic increases of the 4.0 MHz signal. (Note: all spectra were simulated using the EasySpin package (75), version 5.1). *C*, the ^15^N energy level diagram shows observed transitions from coupling to a single ^15^N His, ν_α_ and ν_β_, and transitions from two ^15^N His, revealing an additional signal at 2ν_α_. *D*, ESEEM spectra showing how the 2ν_α_ arises only with more than one coupled His residue. *E*, the two-dimensional HYSCORE Cu^2+^ spectrum resulting from four coordinated ^15^N-His. Cross peaks arise at multiples of ν_α_. ESEEM, electron spin echo envelope modulation; HYSCORE, hyperfine sublevel correlation.

ESEEM, first demonstrated by Mims *et al*. in their development of pulsed EPR (61), continues to find new applications in both electronic materials and biological research. If there are weak nuclear couplings to a paramagnetic center, sweeping out pulse delays in a two or three pulse spin echo sequence will generate time-dependent oscillations of the echo intensity related to the coupled nuclear frequencies (48, 62, 63). As discovered by Peisach and Mims, Cu^2+^-imidazole samples probed at 9 to 10 GHz give pronounced ESEEM modulations with frequencies labeled ν_−_, ν_0_ and ν_+_, approximately matching the ^14^N (I = 1) quadrupolar transitions (64, 65, 66). Moreover, the ^14^N coupling is from the remote, non-coordinated, imidazole nitrogen, which is unique to histidine residues. Consequently, the method is incredibly valuable for identifying His coordination to copper centers, as are typically found in neurodegenerative proteins. ^15^N (I = ½) His also produces pronounced ESEEM signals but with transitions distinct from those of ^14^N His (65). Specifically, the ^15^N nucleus exists in one of two spin states that, when mixed with ^15^N hyperfine coupling, gives rise to two observable transitions labeled ν_α_ and ν_β_. Energy level diagrams and simulated spectra for copper coordinated to ^14^N or ^15^N His, with typical coupling parameters, are shown in Figure 2. The detailed ESEEM theories for these cases are well described elsewhere (48, 63).

Beyond the assignment of His imidazoles coordinated to a copper center, ESEEM also yields spectroscopic features related to the number of coupled nitrogen nuclei. Importantly, the nature of these signals is distinct for each of the two nitrogen isotopes. For ^14^N, multiple His coordination results in a dramatic increase in the intensity of so-called “double quantum” signal at approximately 4.0 MHz (Fig. 2) (23, 64). By contrast, multiple ^15^N His gives rise to an additional signal at 2ν_α_, arising from the transition between the |½, ½> and the |-½, -½> states, as recently characterized by Smart *et al*. (67, 68). The two-dimensional version of ESEEM, referred to as hyperfine sublevel correlation spectroscopy (HYSCORE), further resolves the 2ν_α_ signal as a separate peak (Fig. 2) (67). Moreover, additional ^15^N couplings are evident as further multiples of the fundamental ν_α_ frequency. Given the potential value of the 2ν_α_ ESEEM peak, Smart *et al*. evaluated the timing in three-pulse experiment to simultaneously optimize this signal but without loss of the fundamental ^14^N and ^15^N transitions (67).

## Copper regulation of prion protein neurotoxicity

**Figure 3:**
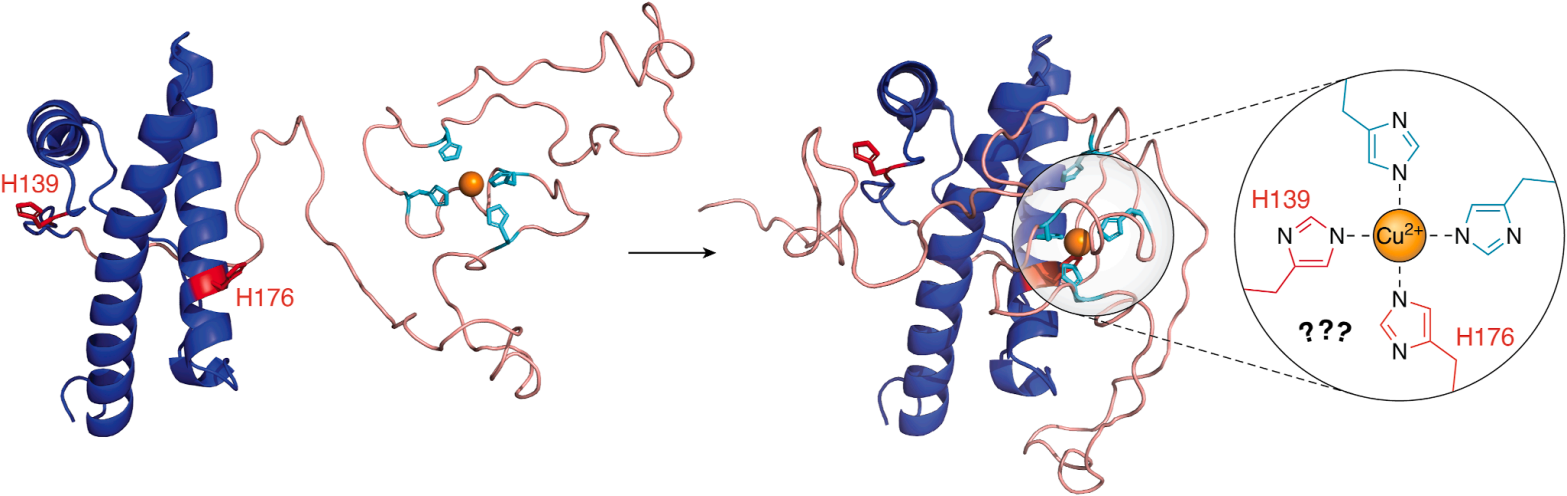
**Identification of His residues available to coordinate Cu^2+^.** Previous work suggested that Cu^2+^ formed a coordination link bringing together the two PrP^C^ domains, shifting the equilibrium to the *right*, tempering the protein’s intrinsic neurotoxicity. However, as indicated by the inset, the specific His residues involved in this regulatory mechanism, as well as the number of His residues from the respective domains, remained unclear. PrP^C^, cellular isoform of prion protein residues 23 to 230.

Figure 3 highlights the two distinct domains of PrP^C^, the predominantly helical C-terminal domain and the extended N-terminal IDR with an equivalent of Cu^2+^ coordinated by the OR His residues. Deletion of specific polypeptide segments between the OR and the C-terminal domain are highly toxic, leading to neonatal death in transgenic mice and spontaneous transmembrane currents in cultured cells and primary neurons (37, 69, 70). Consequently, it is now hypothesized that the toxicity of monomeric PrP^C^ arises from its N terminus, referred to as the “toxic effector” domain (37, 71). Similar N-terminally driven toxic responses may be generated by treatment of WT PrP^C^ with monoclonal antibodies that target specific epitopes on the C-terminal domain (71). The C-terminal domain, therefore, regulates the N-terminal toxic effector domain. Moreover, Cu^2+^ is required for this regulatory interaction since the addition of copper-pentaglycine to a toxic PrP^C^ deletion mutant abrogates toxic transmembrane ionic currents (37).

The PrP^C^ C-terminal regulatory domain possesses two His residues at positions 139 and 176. NMR experiments suggest that the Cu^2+^-occupied OR docks to a C-terminal surface encompassing these two His residues (39). Moreover, simultaneous deletion of both His residues leads to enhanced spontaneous currents in cultured neuroblastoma cells (39). These observations suggest that the regulatory interaction involves copper linking the two PrP^C^ domains through mutual His coordination, as shown schematically in Figure 3 (39, 72). However, the identity of the specific C-terminal His and the respective numbers of C-terminal *versus* OR His remained unknown.

**Figure 4:**
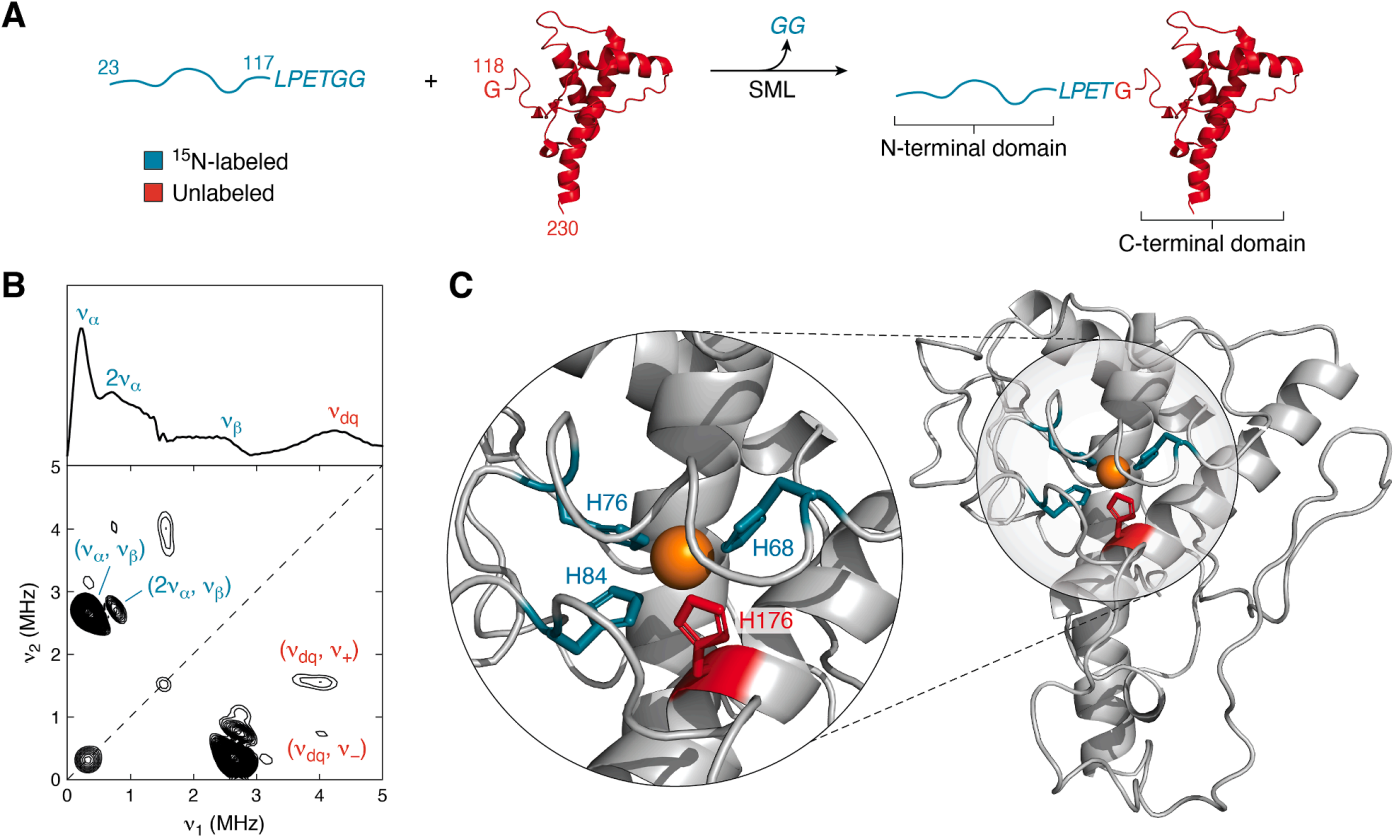
**Strategy for segmental labeling of PrP^C^ and resulting pulsed EPR spectra.***A*, sortase-mediated ligation results in PrP^C^ with uniform ^15^N labeling of residues 23 to 117. *B*, ESEEM (*top*) and HYSCORE (*bottom*) show a superposition of ^14^N- and ^15^N-His Cu^2+^ spectra. The presence of the 2ν_α_ peak indicates multiple N-terminal His at the copper center (detailed analysis in ref (68)). *C*, AlphaFold 3 model showing how Cu^2+^ coordinates simultaneously to H176 of the globular domain and three histidines of the N-terminal OR domain. PrP^C^, cellular isoform of prion protein residues 23 to 230; EPR, electron paramagnetic resonance; ESEEM, electron spin echo envelope modulation; HYSCORE, hyperfine sublevel correlation; OR, octapeptide repeat.

By traditional EPR methods, His coordination to Cu^2+^ centers gives signals with little variation; there is no way to distinguish one ^14^N His from another. However, the unique ^14^N *versus* ^15^N His ESEEM spectra suggested that selective placement of isotopically distinct histidines in a segmentally labeled PrP^C^ construct would critically test for mutual copper coordination between the two domains. As demonstrated by Pavlovici *et al*., segmental labeling was achieved through sortase-mediated ligation (47), as shown in Figure 4 (68). The N-terminal domain through residue 117 was expressed with uniform ^15^N labeling and ligated to the unlabeled C-terminal domain. The resulting construct retained ^14^N at His positions 139 and 176.

ESEEM and HYSCORE of segmentally isotopically labeled PrP^C^ with one equivalent of copper clearly show simultaneous signals of both ^14^N and ^15^N-His (68). In addition, both spectra exhibit 2ν_α_ signals indicating coordination from multiple OR His residues. By contrast, analysis of the ^14^N 4.0 MHz peak suggests coordination by a single C-terminal His residue. Follow up mutagenesis studies identified His 176 as a critical participant in copper coordination and, hence, regulation of the otherwise toxic N-terminal domain. AlphaFold 3 simulations with a single Cu^2+^ ion produced a structure consistent with our EPR experiments (Fig. 4) (68).

## Copper anchors Aβ to PrP^C^ through mutual copper coordination

Accumulation of intraneuronal Aβ (iAβ) is emerging as a distinct contributor to AD perhaps paralleling the more conventional neurodegenerative mechanism of extracellular Aβ aggregation and plaque formation (73, 74). iAβ may associate at organelles such as the mitochondria, ultimately compromising cellular function. Experiments with cultured cells demonstrates that PrP^C^ transports fluorescently-labeled Aβ across the plasma membrane through endocytosis (40). Given that both PrP^C^ and Aβ exhibit high affinity copper binding, it is reasonable to explore whether the prion protein’s Cu^2+^ center provides an anchor point for binding Aβ.

**Figure 5:**
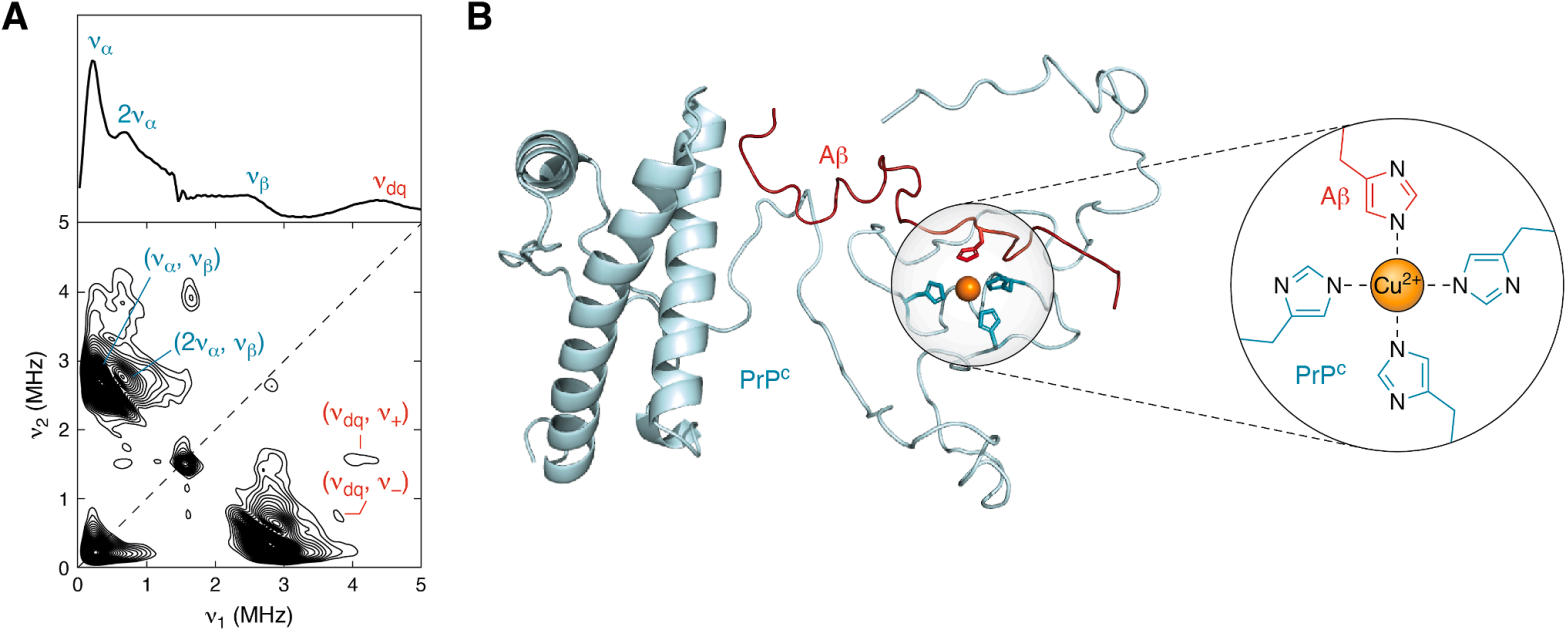
**Isotope-edited ESEEM and HYSCORE of the PrP^C^-Cu^2+^-Aβ complex.** PrP^C^ was uniformly ^15^N-labeled, while Aβ is unlabeled. *A*, ESEEM and HYSCORE of a 1:1:1 mixture reveals the ^15^N 2ν_α_ peak, consistent with multiple prion protein His residues, while the 4.0 MHz peak from ^14^N is clearly present but weak relative to the Cu^2+^-Aβ complex alone. A thorough analysis of these spectra (67) suggests the model in (*B*) where Cu^2+^ anchors the two proteins together, with Aβ (*red*) contributing a single His to the copper center. ESEEM, electron spin echo envelope modulation; HYSCORE, hyperfine sublevel correlation; PrP^C^, cellular isoform of prion protein residues 23 to 230; Aβ, amyloid-β peptide.

Isotope-edited ESEEM was applied to uniformly ^15^N-labeled PrP^C^ in combination with unlabeled Aβ (1, 2, 3, 4, 5, 6, 7, 8, 9, 10, 11, 12, 13, 14, 15, 16, 17, 18, 19, 20, 21, 22, 23, 24, 25, 26, 27, 28, 29, 30), a nonaggregating segment of full-length Aβ (67). Each protein yields a distinct ESEEM spectrum, as shown previously in reference (67). Aβ alone with one equivalent of copper gives a pronounced 4.0 MHz signal indicating multiple His coordination, consistent with participation of the peptide’s multiple His residues. The ESEEM spectrum of ^15^N-PrP^C^ exhibits the 2ν_α_ signal indicating multiple His coordination; ESEEM of the ternary mixture of 1:1:1 PrP^C^, Aβ and Cu^2+^ shows the simultaneous signals of both isotopes (Fig. 5). These features are also present in the companion HYSCORE spectrum (Fig. 5). However, the ^14^N 4.0 MHz ESEEM signal in the ternary mixture is greatly reduced relative to that from Aβ alone. Along with persistence of the 2ν_α_ transition, these findings point to a coordination environment composed of multiple His from PrP^C^ but only a single His from Aβ, as shown schematically in Figure 5 (67). Reduction of the copper concentration to below one equivalent in the otherwise 1:1 PrP^C^:Aβ mixture results in ESEEM spectra equivalent to that observed in Figure 5. This suggests that the ternary complex is thermodynamically stable, thus providing compelling evidence that the coordination model in Figure 5 represents a plausible complex for PrP^C^-mediated Aβ transport.

These experiments provide a molecular perspective, and testable hypotheses, for how the prion protein interacts with Aβ, as necessary for PrP^C^-mediated Aβ endocytosis. Aβ transport by PrP^C^ endocytosis might be part of normal cellular physiology in which the peptide is ultimately degraded through the endo-lysosome pathway. However, it is also plausible that high levels of extracellular Aβ could overwhelm this degradation pathway, ultimately leading to the accumulation of iAβ. Elucidation of the relevant Aβ pathways, along with further clarification of the PrP^C^:Aβ:Cu^2+^ complex could provide a new level of understanding and identification of therapeutic targets for treating AD.

## Conclusions

ESEEM and HYSCORE are ideal for assessing structural details in IDR-Cu^2+^ complexes, relevant to neurodegenerative proteins. With isotope-edited pulsed EPR, we have expanded the capabilities of these robust methods providing new insights into both the regulation of PrP^C^ toxicity and PrP^C^-mediated Aβ transport. It is our hope that the methods reviewed here contribute to the development of new concepts and strategies for treating the neurodegenerative maladies that plague our aging population.

## Conflict of interest

The authors declare that they have no conflicts of interest with the contents of this article.

## References

[1] Selkoe D.J., Hardy J. (2016). The amyloid hypothesis of Alzheimer's disease at 25 years. EMBO Mol. Med..

[2] Brody A.H., Strittmatter S.M. (2018). Synaptotoxic signaling by amyloid beta oligomers in Alzheimer's disease through prion protein and mGluR5. Adv. Pharmacol..

[3] DeTure M.A., Dickson D.W. (2019). The neuropathological diagnosis of Alzheimer's disease. Mol. Neurodegener..

[4] Zhang H., Wei W., Zhao M., Ma L., Jiang X., Pei H. (2021). Interaction between abeta and tau in the pathogenesis of Alzheimer's disease. Int. J. Biol. Sci..

[5] Wang H., Wang M., Wang B., Li M., Chen H., Yu X. (2012). Immunogold labeling and X-ray fluorescence microscopy reveal enrichment ratios of Cu and Zn, metabolism of APP and amyloid-beta plaque formation in a mouse model of Alzheimer's disease. Metallomics.

[6] Zhu X., Victor T.W., Ambi A., Sullivan J.K., Hatfield J., Xu F. (2020). Copper accumulation and the effect of chelation treatment on cerebral amyloid angiopathy compared to parenchymal amyloid plaques. Metallomics.

[7] Deibel M.A., Ehmann W.D., Markesbery W.R. (1996). Copper, iron, and zinc imbalances in severely degenerated brain regions in Alzheimer's disease: possible relation to oxidative stress. J. Neurol. Sci..

[8] Xu J., Church S.J., Patassini S., Begley P., Waldvogel H.J., Curtis M.A. (2017). Evidence for widespread, severe brain copper deficiency in Alzheimer's dementia. Metallomics.

[9] Bourassa M.W., Leskovjan A.C., Tappero R.V., Farquhar E.R., Colton C.A., Van Nostrand W.E. (2013). Elevated copper in the amyloid plaques and iron in the cortex are observed in mouse models of Alzheimer's disease that exhibit neurodegeneration. Biomed. Spectrosc. Imaging.

[10] Lovell M.A., Robertson J.D., Teesdale W.J., Campbell J.L., Markesbery W.R. (1998). Copper, iron and zinc in Alzheimer's disease senile plaques. J. Neurol. Sci..

[11] Miller L.M., Wang Q., Telivala T.P., Smith R.J., Lanzirotti A., Miklossy J. (2006). Synchrotron-based infrared and X-ray imaging shows focalized accumulation of Cu and Zn co-localized with beta-amyloid deposits in Alzheimer's disease. J. Struct. Biol..

[12] Pushie M.J., Pickering I.J., Martin G.R., Tsutsui S., Jirik F.R., George G.N. (2011). Prion protein expression level alters regional copper, iron and zinc content in the mouse brain. Metallomics.

[13] Okafor M., Faller P., Vitale N. (2025). Cell-specific copper dyshomeostasis mechanism in Alzheimer's disease. Transl Neurodegener.

[14] Zhong G., Wang X., Li J., Xie Z., Wu Q., Chen J. (2024). Insights into the role of copper in neurodegenerative diseases and the therapeutic potential of natural compounds. Curr. Neuropharmacol..

[15] Bagheri S., Squitti R., Haertle T., Siotto M., Saboury A.A. (2017). Role of copper in the onset of Alzheimer's disease compared to other metals. Front. Aging Neurosci..

[16] Alies B., Renaglia E., Rozga M., Bal W., Faller P., Hureau C. (2013). Cu(II) affinity for the Alzheimer's peptide: tyrosine fluorescence studies revisited. Anal. Chem..

[17] Karr J.W., Kaupp L.J., Szalai V.A. (2004). Amyloid-beta binds Cu2+ in a mononuclear metal ion binding site. J. Am. Chem. Soc..

[18] Syme C.D., Nadal R.C., Rigby S.E., Viles J.H. (2004). Copper binding to the amyloid-beta (abeta) peptide associated with Alzheimer's disease: folding, coordination geometry, pH dependence, stoichiometry, and affinity of Abeta-(1-28): insights from a range of complementary spectroscopic techniques. J. Biol. Chem..

[19] Summers K.L., Roseman G.P., Sopasis G.J., Millhauser G.L., Harris H.H., Pickering I.J. (2020). Copper(II) binding to PBT2 differs from that of other 8-Hydroxyquinoline chelators: implications for the treatment of neurodegenerative protein misfolding diseases. Inorg. Chem..

[20] Hong L., Carducci T.M., Bush W.D., Dudzik C.G., Millhauser G.L., Simon J.D. (2010). Quantification of the binding properties of Cu2+ to the amyloid beta peptide: coordination spheres for human and rat peptides and implication on Cu2+-induced aggregation. J. Phys. Chem. B.

[21] Quintanar L., Millhauser G.L. (2022). EPR of copper centers in the prion protein. Methods Enzymol..

[22] Millhauser G.L. (2007). Copper and the prion protein: methods, structures, function, and disease. Annu. Rev. Phys. Chem..

[23] Chattopadhyay M., Walter E.D., Newell D.J., Jackson P.J., Aronoff-Spencer E., Peisach J. (2005). The octarepeat domain of the prion protein binds Cu(II) with three distinct coordination modes at pH 7.4. J. Am. Chem. Soc..

[24] Millhauser G.L. (2004). Copper binding in the prion protein. Acc. Chem. Res..

[25] Burns C.S., Aronoff-Spencer E., Legname G., Prusiner S.B., Antholine W.E., Gerfen G.J. (2003). Copper coordination in the full-length, recombinant prion protein. Biochemistry.

[26] Burns C.S., Aronoff-Spencer E., Dunham C.M., Lario P., Avdievich N.I., Antholine W.E. (2002). Molecular features of the copper binding sites in the octarepeat domain of the prion protein. Biochemistry.

[27] Aronoff-Spencer E., Burns C.S., Avdievich N.I., Gerfen G.J., Peisach J., Antholine W.E. (2000). Identification of the Cu^2+^ binding sites in the N-terminal domain of the prion protein by EPR and CD spectroscopy. Biochemistry.

[28] Legname G. (2023). Copper coordination modulates prion conversion and infectivity in mammalian prion proteins. Prion.

[29] Dudzik C.G., Walter E.D., Abrams B.S., Jurica M.S., Millhauser G.L. (2013). Coordination of copper to the membrane-bound form of alpha-synuclein. Biochemistry.

[30] Uversky V.N., Li J., Fink A.L. (2001). Metal-triggered structural transformations, aggregation, and fibrillation of human alpha-synuclein. A possible molecular NK between Parkinson's disease and heavy metal exposure. J. Biol. Chem..

[31] Rasia R.M., Bertoncini C.W., Marsh D., Hoyer W., Cherny D., Zweckstetter M. (2005). Structural characterization of copper(II) binding to alpha-synuclein: insights into the bioinorganic chemistry of Parkinson's disease. Proc. Natl. Acad. Sci. U. S. A..

[32] Maryon E.B., Molloy S.A., Zimnicka A.M., Kaplan J.H. (2007). Copper entry into human cells: progress and unanswered questions. Biometals.

[33] Faller P., Hureau C. (2009). Bioinorganic chemistry of copper and zinc ions coordinated to amyloid-beta peptide. Dalton Trans..

[34] Prusiner S.B. (2013). Biology and genetics of prions causing neurodegeneration. Annu. Rev. Genet..

[35] Evans E.G.B., Millhauser G.L. (2017). Copper- and zinc-promoted interdomain structure in the prion protein: a mechanism for autoinhibition of the neurotoxic N-Terminus. Prog. Mol. Biol. Transl. Sci..

[36] Evans E.G.B., Pushie M.J., Markham K.A., Lee H.-W., Millhauser G.L. (2016). Interaction between prion protein's copper-bound octarepeat domain and a charged C-Terminal pocket suggests a mechanism for N-Terminal regulation. Structure.

[37] Wu B., McDonald A.J., Markham K., Rich C.B., McHugh K.P., Tatzelt J. (2017). The N-terminus of the prion protein is a toxic effector regulated by the C-terminus. eLife.

[38] McDonald A.J., Leon D.R., Markham K.A., Wu B., Heckendorf C.F., Schilling K. (2019). Altered domain structure of the prion protein caused by Cu2+ binding and functionally relevant mutations: analysis by cross-linking, MS/MS, and NMR. Structure.

[39] Schilling K.M., Tao L., Wu B., Kiblen J.T.M., Ubilla-Rodriguez N.C., Pushie M.J. (2020). Both N-Terminal and C-terminal histidine residues of the prion protein are essential for copper coordination and neuroprotective self-regulation. J. Mol. Biol..

[40] Foley A.R., Roseman G.P., Chan K., Smart A., Finn T.S., Yang K. (2020). Evidence for aggregation-independent, PrP^c^-mediated abeta cellular internalization. Proc. Natl. Acad. Sci. U. S. A..

[41] Lauren J., Gimbel D.A., Nygaard H.B., Gilbert J.W., Strittmatter S.M. (2009). Cellular prion protein mediates impairment of synaptic plasticity by amyloid-beta oligomers. Nature.

[42] Smith L.M., Strittmatter S.M. (2017). Binding sites for amyloid-beta oligomers and synaptic toxicity. Cold Spring Harb. Perspect. Med..

[43] Haas L.T., Salazar S.V., Kostylev M.A., Um J.W., Kaufman A.C., Strittmatter S.M. (2016). Metabotropic glutamate receptor 5 couples cellular prion protein to intracellular signalling in Alzheimer's disease. Brain.

[44] Gouras G.K., Almeida C.G., Takahashi R.H. (2005). Intraneuronal abeta accumulation and origin of plaques in Alzheimer's disease. Neurobiol. Aging.

[45] Ferrer I., Blanco R., Carmona M., Puig B., Ribera R., Rey M.J. (2001). Prion protein expression in senile plaques in Alzheimer's disease. Acta Neuropathol..

[46] Nieznanski K., Surewicz K., Chen S., Nieznanska H., Surewicz W.K. (2014). Interaction between prion protein and Abeta amyloid fibrils revisited. ACS Chem. Neurosci..

[47] Mao H., Hart S.A., Schink A., Pollok B.A. (2004). Sortase-mediated protein ligation: a new method for protein engineering. J. Am. Chem. Soc..

[48] Dikanov S.A., Tsvetkov Y.D. (1992).

[49] Zahn R., Liu A., Luhrs T., Riek R., von Schroetter C., Lopez Garcia F. (2000). NMR solution structure of the human prion protein. Proc. Natl. Acad. Sci. U. S. A..

[50] Walter E.D., Stevens D.J., Spevacek A.R., Visconte M.P., Dei Rossi A., Millhauser G.L. (2009). Copper binding extrinsic to the octarepeat region in the prion protein. Curr. Protein Pept. Sci..

[51] Torres J.M., Marin-Moreno A., Espinosa J.C., Canoyra S., Burato A., Ciullini A. (2025). Substitution of histidine 95 by tyrosine in the prion protein causes spontaneous neurodegeneration in transgenic mice. PLoS Pathog..

[52] Karr J.W., Akintoye H., Kaupp L.J., Szalai V.A. (2005). N-Terminal deletions modify the Cu2+ binding site in amyloid-beta. Biochemistry.

[53] Crnich E., Lullo R., Tabaka A., Havens M.A., Kissel D.S. (2021). Interactions of copper and copper chelate compounds with the amyloid beta peptide: an investigation into electrochemistry, reactive oxygen species and peptide aggregation. J. Inorg. Biochem..

[54] Guilloreau L., Damian L., Coppel Y., Mazarguil H., Winterhalter M., Faller P. (2006). Structural and thermodynamical properties of CuII amyloid-beta16/28 complexes associated with Alzheimer's disease. J. Biol. Inorg. Chem..

[55] Shin B.K., Saxena S. (2008). Direct evidence that all three histidine residues coordinate to Cu(II) in amyloid-beta1-16. Biochemistry.

[56] Summers K.L., Roseman G., Schilling K.M., Dolgova N.V., Pushie M.J., Sokaras D. (2022). Alzheimer's drug PBT2 interacts with the amyloid beta 1-42 peptide differently than other 8-Hydroxyquinoline chelating drugs. Inorg. Chem..

[57] Mantri Y., Fioroni M., Baik M.H. (2008). Computational study of the binding of CuII to Alzheimer's amyloid-beta peptide: do Abeta42 and Abeta40 bind copper in identical fashion?. J. Biol. Inorg. Chem..

[58] Squitti R., Faller P., Hureau C., Granzotto A., White A.R., Kepp K.P. (2021). Copper imbalance in Alzheimer's disease and its link with the amyloid hypothesis: towards a combined clinical, chemical, and genetic etiology. J. Alzheimers Dis..

[59] Camerman N., Camerman A., Sarkar B. (1976). Molecular design to mimic the copper(II) transport site of human albumin. The crystal and molecular structure of copper(II)-glycylglycyl-L-histidine-N-methyl amide monoaquo complex. Can. J. Chem..

[60] Deschamps P., Kulkarni P.P., Sarkar B. (2004). X-ray structure of physiological copper(II)-bis(L-histidinato) complex. Inorg. Chem..

[61] Mims W.B., Nassau K., McGee J.D. (1961). Spectral diffusion in electron resonance lines. Phys. Rev..

[62] Cutsail G.E., Telser J., Hoffman B.M. (2015). Advanced paramagnetic resonance spectroscopies of iron-sulfur proteins: electron nuclear double resonance (ENDOR) and electron spin echo envelope modulation (ESEEM). Biochim. Biophys. Acta.

[63] Schweiger A., Jeschke G. (2005).

[64] Mccracken J., Pember S., Benkovic S.J., Villafranca J.J., Miller R.J., Peisach J. (1988). Electron-spin echo studies of the copper-binding site in phenylalanine-hydroxylase from chromobacterium-violaceum. J. Am. Chem. Soc..

[65] Mims W.B., Peisach J. (1978). The nuclear modulation effect in electron spin echoes for complexes of Cu^2+^ and imidazole with ^14^N and ^15^N. J. Chem. Phys..

[66] Mims W.B., Peisach J., Berliner L.J., Reuben J. (1981). Biological Magnetic Resonance.

[67] Smart A., Singewald K., Hasanbasri Z., Britt R.D., Millhauser G.L. (2025). Identifying the copper coordination environment between interacting neurodegenerative proteins: a new approach using pulsed EPR with (14)N/(15)N isotopic labeling. J. Biol. Chem..

[68] Pavlovici F.A., Singewald K., Kaplan S., Chen E., Millhauser G.L. (2025). Segmental isotope labeling of the prion protein: identification of a key residue for copper-mediated interdomain structure. ACS Chem. Biol..

[69] Baumann F., Tolnay M., Brabeck C., Pahnke J., Kloz U., Niemann H.H. (2007). Lethal recessive myelin toxicity of prion protein lacking its central domain. EMBO J..

[70] Li A., Christensen H.M., Stewart L.R., Roth K.A., Chiesa R., Harris D.A. (2007). Neonatal lethality in transgenic mice expressing prion protein with a deletion of residues 105–125. EMBO J..

[71] Sonati T., Reimann R.R., Falsig J., Baral P.K., O'Connor T., Hornemann S. (2013). The toxicity of antiprion antibodies is mediated by the flexible tail of the prion protein. Nature.

[72] Schilling K.M., Jorwal P., Ubilla-Rodriguez N.C., Assafa T.E., Gatdula J.R.P., Harris D.A. (2023). N-Glycosylation is a potent regulator of prion protein neurotoxicity. J. Biol. Chem..

[73] Brewer G.J., Herrera R.A., Philipp S., Sosna J., Reyes-Ruiz J.M., Glabe C.G. (2020). Age-related intraneuronal aggregation of amyloid-beta in endosomes, mitochondria, autophagosomes, and lysosomes. J. Alzheimers Dis..

[74] Welikovitch L.A., Do Carmo S., Magloczky Z., Malcolm J.C., Loke J., Klein W.L. (2020). Early intraneuronal amyloid triggers neuron-derived inflammatory signaling in APP transgenic rats and human brain. Proc. Natl. Acad. Sci. U. S. A..

[75] Stoll S., Schweiger A. (2006). EasySpin, a comprehensive software package for spectral simulation and analysis in EPR. J. Magn. Reson..

